# Peptide Functionalized Nanoplasmonic Sensor for Explosive Detection

**DOI:** 10.1007/s40820-015-0059-z

**Published:** 2015-08-14

**Authors:** Diming Zhang, Qian Zhang, Yanli Lu, Yao Yao, Shuang Li, Jing Jiang, Gang Logan Liu, Qingjun Liu

**Affiliations:** 1 Biosensor National Special Laboratory, Key Laboratory for Biomedical Engineering of Education Ministry, Department of Biomedical Engineering, Zhejiang University, Hangzhou, 310027 People’s Republic of China; 2 Micro and Nanotechnology Lab, University of Illinois at Urbana-Champaign, Champaign, IL 61801 USA

**Keywords:** Nanocup arrays, Peptide, 2,4,6-trinitrotoluene (TNT), Localized surface plasmon resonance (LSPR), Nanosensor

## Abstract

In this study, a nanobiosensor for detecting explosives was developed, in which the peptide was synthesized with trinitrotoluene (TNT)-specific sequence and immobilized on nanodevice by Au–S covalent linkage, and the nanocup arrays were fabricated by nanoimprint and deposited with Au nanoparticles to generate localized surface plasmon resonance (LSPR). The device was used to monitor slight change from specific binding of 2,4,6-TNT to the peptide. With high refractive index sensing of ~10^4^ nm/RIU, the nanocup device can detect the binding of TNT at concentration as low as 3.12 × 10^−7^ mg mL^−1^ by optical transmission spectrum modulated by LSPR. The nanosensor is also able to distinguish TNT from analogs of 2,4-dinitrotoluene and 3-nitrotoluene in the mixture with great selectivity. The peptide-based nanosensor provides novel approaches to design versatile biosensor assays by LSPR for chemical molecules.

## Introduction

Explosive detections, especially for 2,4,6-trinitrotoluene (TNT), were of sustainable importance due to their threats for public security and human health as pollutants in natural water, soil, and air [1–3]. Thus, over the last couple of decades, significant efforts have been made to develop sensor devices which can detect explosive compounds rapidly, selectively, and sensitively [4–6]. These devices often used explosive sensitive materials, such as molecularly imprinted polymers, carbon nanotubes, and antibodies, to modify transducers, ranging from electrodes to fluorescent assays, for specific explosive detections. With a series of work, these devices have been demonstrated to discern explosive molecules with high sensitivity and selectivity in complex environment. Among them, biosensors attracted increasing focus because of their excellent performances in explosive detections, which might come from biological olfactory perception [7–9]. In recent studies, researches attempted to gradually integrate bio-inspired components, including whole cells, proteins, and aptamers, into various sensor platforms to memetic sniffer abilities of animals, providing a good option to replace sniffer animals in practical explosive detections.

In biosensor studies, proteins such as enzymes, antigens–antibodies, and receptors were common biosensing elements to bind target molecules and then elicit sensor responses in biochemical detections [10–12]. The proteins often had binding sites in which multiple interactions can be formed with target molecules by high specific affinities or catalysis activities. However, these complex proteins were difficult to purify and synthesize, which hindered their biosensing applications. In recent studies, peptides were widely applied as alternative biosensing materials for complex proteins in biosensor designs [13–15]. Peptides were short chains of amino acids that can be designed based on binding sites of proteins, screened into specific sequences, and finally synthesized with chemical methods. It provided an easy option to design artificial receptors to mimic molecular recognitions between proteins and analytes. The robust structures of peptides also allowed for using in more extreme environment and long-term storage, compared to natural protein molecules. Thus, peptides were ideal candidates of biosensing materials for biosensor fabrications.

However, without folding structures, peptides usually elicited slight changes in the interactions with small target molecules, which proposed high sensitivity demand for sensor methods. Recently, nanomaterials (e.g., nanoparticles, carbon nanotubes, nanoholes, and nanowires) became a new focus of ultrasensitive detection in biosensor fields [16–19]. Optical, electrical, and electromechanical methods were applied to record signals from bio-functionalized nanostructures to achieve ultrasensitive detections for various chemicals. Using these nanosensors, bio-interactions could be monitored quantifiably and ultrasensitively, even at single molecular level. Among these platforms, optical detection utilizing plasmon resonance on nanostructures was one of the most common methods for nanoscale biosensors. It was also believed particularly promising because optical measurement allowed remote transduction of biomolecular binding signal without any physical connection between excitation sources and detecting elements. Thus, many biosensors were fabricated depending on plasmon resonance from nanoscale materials, such as localized surface plasmon resonance (LSPR) and surface-enhanced Raman scattering, to analyze chemicals with high sensitivities [20–22].

Here, a kind of TNT-specific peptide was designed and synthesized to modify nanocup arrays (nanoCA) for explosive detections based on LSPR. With refractive index sensing, the nanoCA could monitor binding of TNT to the peptide, while the peptide was immobilized on nanoCA by Au–S covalent linkage. In the measurement, the optical LSPR responses of the peptide-modified devices were recorded with transmission mode in the presence of explosive compounds. TNT, 2,4-Dinitrotoluene (DNT), and 3-Nitrotoluene (3-NT) were also detected at increasing concentrations, respectively. It was demonstrated that the nanosensor could detect TNT selectively in dose-dependence behavior. The mixture of TNT with DNT and 3-NT was used to show high specificity of the nanosensor using synthesized peptide. Thus, this study provided a selective and sensitive nanobiosensor platform for explosive detection.

## Methods and Materials

### Fabrication of NanoCA

**Fig. 1:**
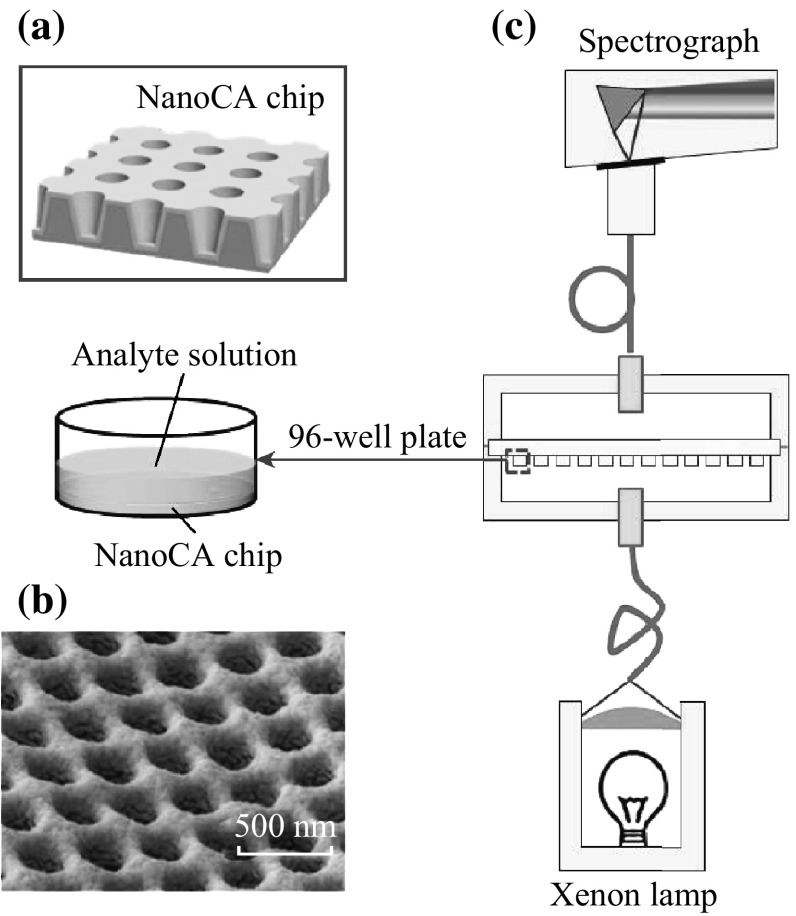
The nanoCA device and its optical measurement system. **a** Structure of periodic nanocups on the nanoCA chip. **b** SEM image of the nanoCA chip. **c** Schematic diagram of the optical detection instrument in transmission mode with a 96-well plate. The *inset* showed individual well integrated nanoCA chip and added with analyte solution

Periodic nanostructures often had outstanding optical features such as absorption and transmission peaks in visible region, which was elicited by plasmon resonance and could be utilized in optical sensors [16]. In this study, hybrid structures of nanocups and nanoparticles were fabricated in arrays to generate LSPR. The fabrication of nanoCA device has been described in our previous reports in detail [23]. In particular, UV curable polymer was evenly distributed on nanocone template, and then 250-μm thick flexible poly (ethylene terephthalate) (PET) sheet was covered on UV curable polymer as supporting substrate. A UV light-curing flood lamp system (EC-Series, Dymax, USA) with average power density of 105 mW cm^−2^ was used to cure the UV polymer for 60 s at room temperature. Thus, nanocups were fabricated in arrays, while the top and bottom diameter of individual cup was fixed at 180 and 100 nm, respectively. The depth of the cup was 500 nm. Figure 1a shows a schematic of nanoCA device. Then, a six-pocket e-beam evaporation system (FC/BJD2000, Temescal, USA) was used for the Au evaporation. Au nanoparticles (20 nm) were deposited on the devices with a thin adhesive layer of Titanium (5 nm). Figure 1b shows nanostructure image obtained by scanning electron microscope (SEM, XL30-ESEM, Philips, Netherlands).

### Optical LSPR Measurement for NanoCA

The optical detection system was multi-mode detection platform (SpectraMax Paradigm, Molecular Devices Co., USA). Normal transmission model was applied to measure the spectra of nanoCA. As shown in Fig. 1c, the nanoCA chips were integrated into 96-well plates for high-throughput measurement. Light was emitted from the light source on the bottom of device, delivered through nanoCA chips, and received by spectrograph. During measurement, 10 μL solution containing detecting compounds was added on the surface of the chip. The small volume of the analyte formed a thin liquid layer to reduce interferences from solution itself. The scanning range was set from 300 to 900 nm and the step was fixed on 1 nm.

### Synthesis and Immobilization of the Peptide

The peptide was chemically synthesized based on the reported TNT-sensitive sequence (WHWQRPLMPVSI) as bio-recognition component for TNT [24]. An aspartic acid was added at the carboxy terminus of the chain as ‘linking residue’ tail. Then, thiol group (–SH) was added at the carboxy terminus of new sequence (WHWQRPLMPVSID) with cysteamide to generate a covalent bond of Au–S on the surface of nanoCA. The chemical synthesis of the peptide was performed with solid phase method by stepwise addition of protected amino acids to a growing peptide chain. The property of the synthesized peptide was tested by high-performance liquid chromatography (HPLC) and mass spectrometry (MS). Before the sensor experiments, the peptide was stored in the form of freeze-dried powders.

For immobilization of the peptide on the nanodevice, the peptide was dissolved in phosphate buffered saline (PBS, pH 7.4) at concentration of 250 μg mL^−1^. 100 μL peptide solution was spotted on the well of microplate. The solution of peptide was diffused and distributed evenly on the device due to the surface tension. After incubated for 12 h, PBS buffer was used to remove the unbonded peptide and nitrogen was used to dry the devices. Then, the nanodevice with peptide was stored under 4 °C and ready for explosive detections. All of the above immobilizing processes were performed at room temperature (22 °C).

### Measurement for Explosive Detection

The standard substances of TNT, DNT, and 3-NT were purchased from Aladdin (Shanghai, China) in methanol solution at concentration of 1 mg mL^−1^. In explosive detections, the standard solution was diluted to five different concentrations (10^−6^, 10^−5^, 10^−4^, 10^−3^, and 10^−2^ mg mL^−1^) with methanol from the original concentration. The transmission spectrums of nanoCA were recorded by the optical system, when 30 μL analyte solution at different concentrations was added into different cavities. The all nanodevices were used once and disposable. Other reagents in experiments were analytical grades which were purchased from Aladdin (Shanghai, China).

## Results

### TNT-Specific Peptide Synthesis

**Fig. 2:**
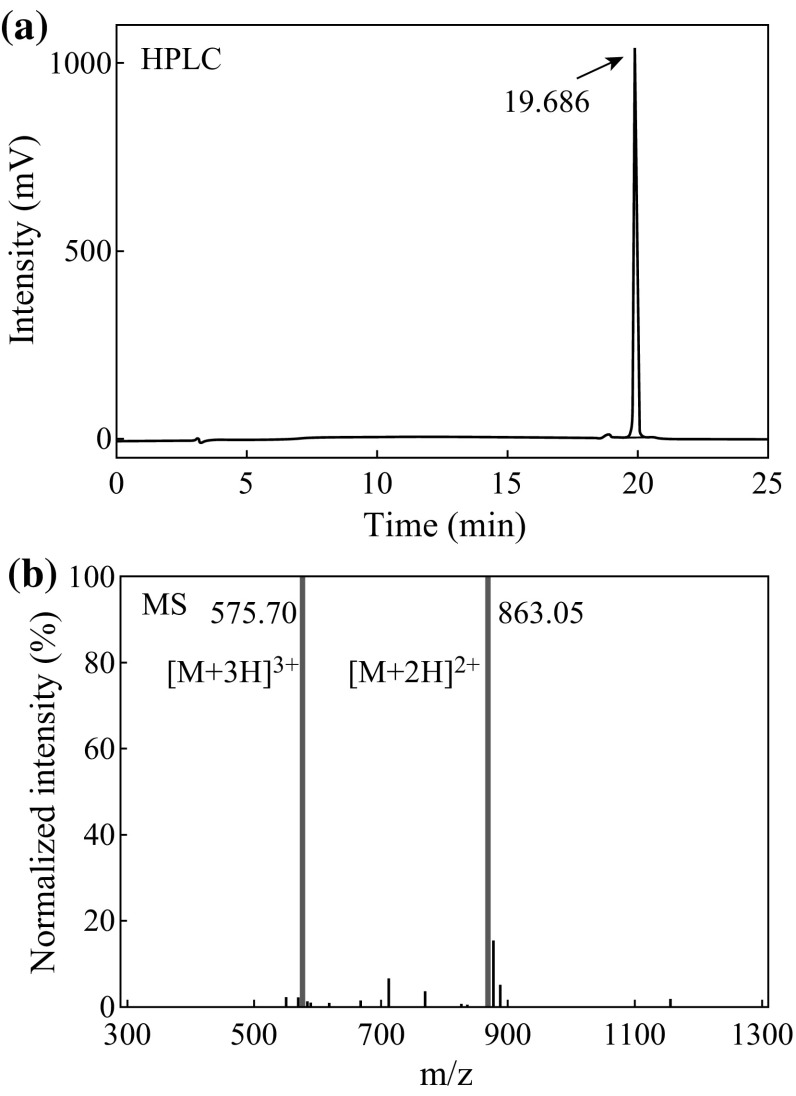
Qualification of the TNT-specific peptide. **a** HPLC test for the synthesized peptide. **b** MS test for the synthesized peptide

The HPLC and MS were used to evaluate the purity of the peptide and to check the peptide sequence, respectively. As shown in Fig. 2a, HPLC showed a significant intensity peak in 19.686 min, which occupied 96 % of all peak area in HPLC. It suggested that the synthesized compounds had a high purity of 96 %. Then, MS was used to determine the molecular weight of the synthesized peptide. As expected, the MS showed two pulses at 575.70 and 863.05 for M+3H^+^ and M+2H^+^, respectively (Fig. 2b). These both indicated that the molecular weight of the peptide was about 1724.1, which was coincident with theoretical value based on amino acid sequences. Thus, with HPLC and MS, it could be found that the peptide was synthesized in reported sequence with high purity and ready for bio-functionalization of the nanosensor.

### Optical Measurement for LSPR Property of the Nanodevice

**Fig. 3:**
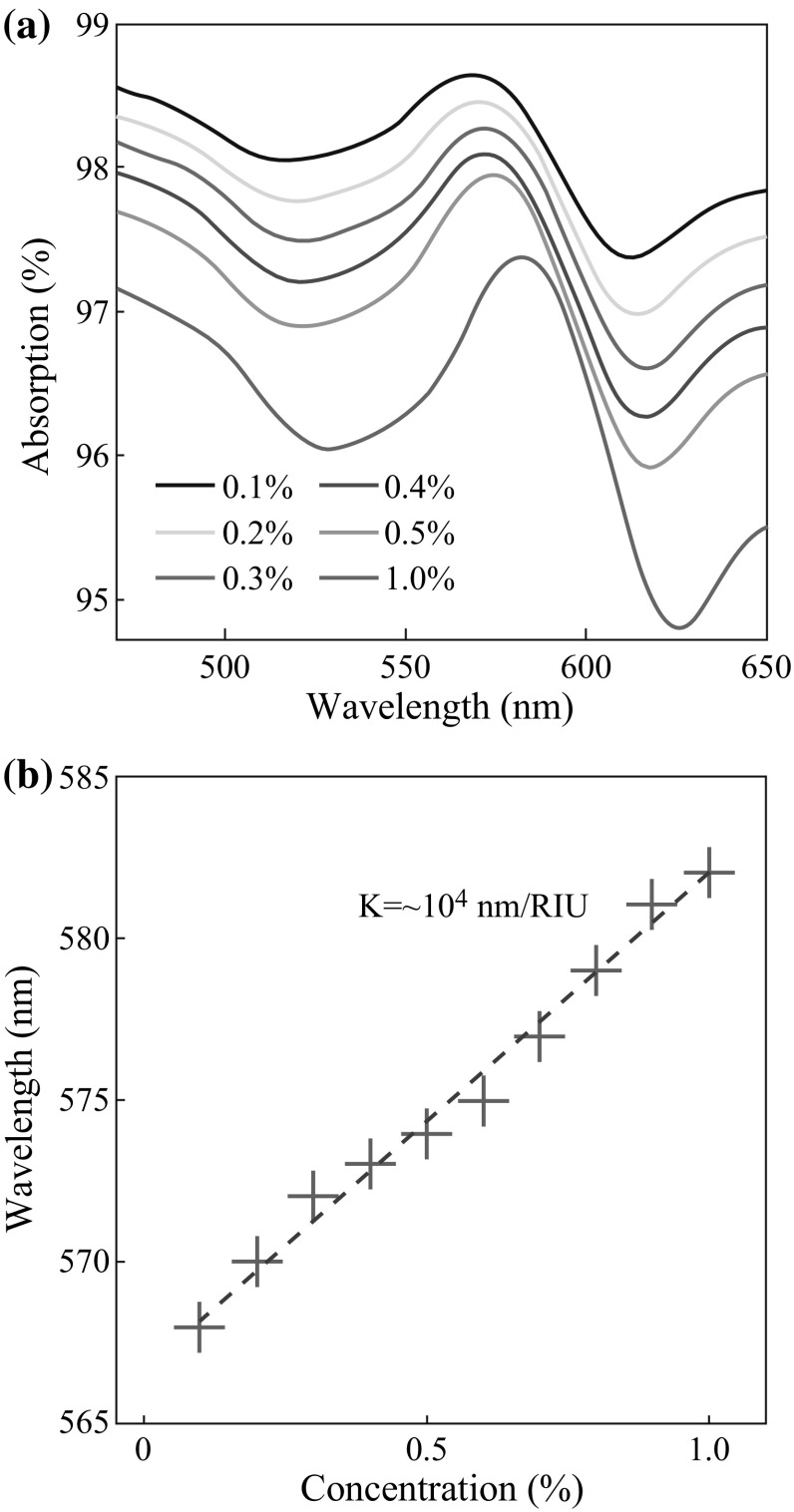
Refractive index sensing property of nanoCA chip. **a** Absorption spectrum of nanoCA with NaCl at increasing concentrations. **b** Statistics for resonance wavelengths from absorption spectrum of nanoCA chips

To investigate the refractive index sensing property of the nanoCA chip, NaCl solution at increasing concentrations was added on the surface of nanoCA chip in the 96-well plate. The increments of concentrations meant the growth of refractive index of environment in which nanocups stayed. As shown in Fig. 3a, significant peaks could be observed in visible range from 500 to 650 nm, when the absorption spectrum through nanoCA chips was recorded. The absorption peaks had right shifts in resonance wavelengths with increment of refractive index. The statistics showed the linear relationship between NaCl concentrations and resonance wavelengths (Fig. 3b). When the concentrations of NaCl solution changed from 0.1 to 1 %, the refractive index change was about 1.5 × 10^−3^ refractive index unit (RIU) and elicited 14 nm shift in resonance wavelength. Thus, the nanoCA device had the sensitivity about ~10^4^ nm per RIU, which showed same good performance as nanostructured sensor devices in several recent researches [25, 26]. It provided a great guarantee to monitor slight changes of peptide in explosive detections.

### Bio-functionalization of the NanoCA with Peptide

**Fig. 4:**
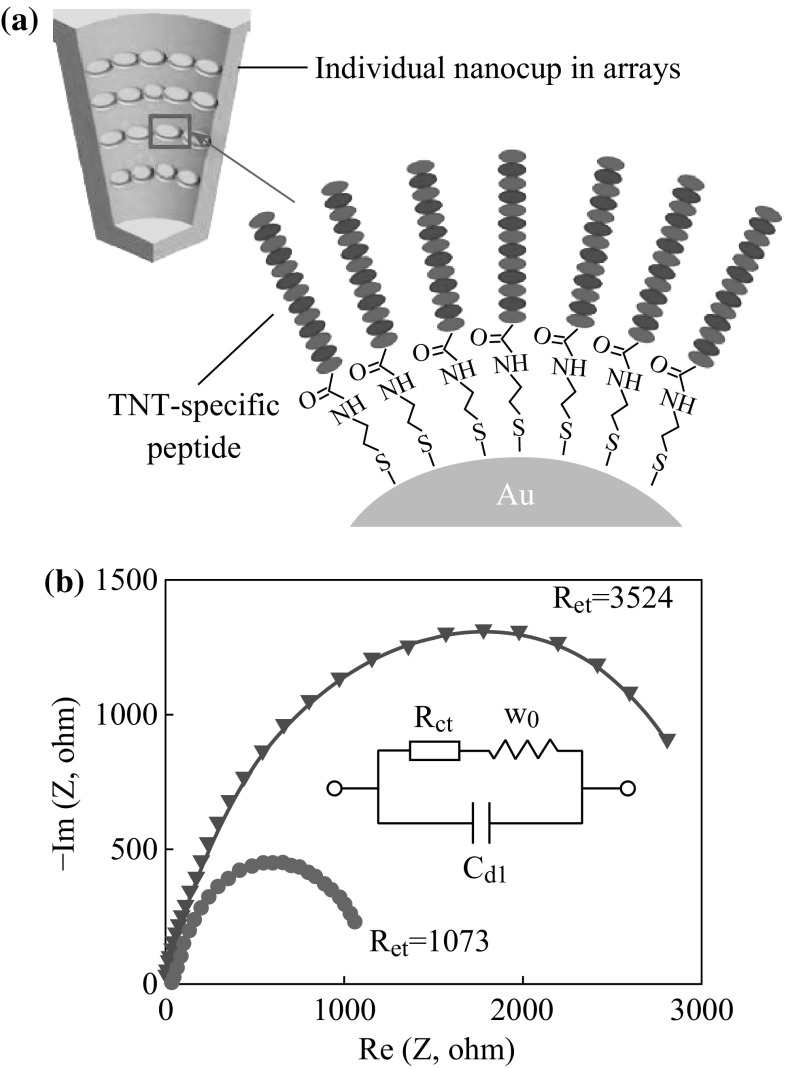
Immobilization of the peptide on the nanoCA device. **a** Schematic of the peptide immobilization on the nanoCA with covalent Au–S linkage (Au nanoparticles are deposited everywhere of nanocups). **b** Nyquist plots for nanoCA with (*blue line*) and without (*red line*) the peptide immobilization. The *inset* showed Randles circuit model that described electrochemical property of the device in electrolyte. (Color figure online)

The immobilization of peptide converted the nanoCA from an optical device into a biosensor for specific analyte detections. In the experiment, the peptide was immobilized on the nanoCA by covalent Au–S linkage directly (Fig. 4a). Compared to other self-assemble methods, this immobilization did not need extra chemical agent and complex operations to form amide coupling, which reduced expense and time cost for the measurement. In our previous report, it has been demonstrated that nanoCA devices could be used as work electrodes in electrochemical measurement [27]. The peptide immobilization would change the whole conductivity of nanoCA device. Thus, electrochemical impedance spectroscopy was used to characterize the immobilization of peptide on the nanoCA. The redox couples of K_4_[Fe(CN)_6_]/K_3_[Fe(CN)_6_] (1:1) were used to enhance and exhibit the electronic activates on the nanoCA surface. In Fig. 4b, the Nyquist plots (Re(*Z*) vs. –Im (*Z*)) show the electrochemical impedance change before and after the peptide immobilization. The nanoCA modified with peptide had significant increasing impedance than the nanoCA without peptide. Especially, a specific value *R*_ct_ could be obtained by the Randles circuit model to represent the interface impedance change from the peptide immobilization [28, 29]. The *R*_ct_ was calculated into 3524 ohm after the peptide immobilization, which increased more than three times than value of 1073 ohm before the immobilization. This suggested efficient immobilization of the peptide on nanoCA for explosive detections.

### Explosive Measurement for Trinitrotoluene

**Fig. 5:**
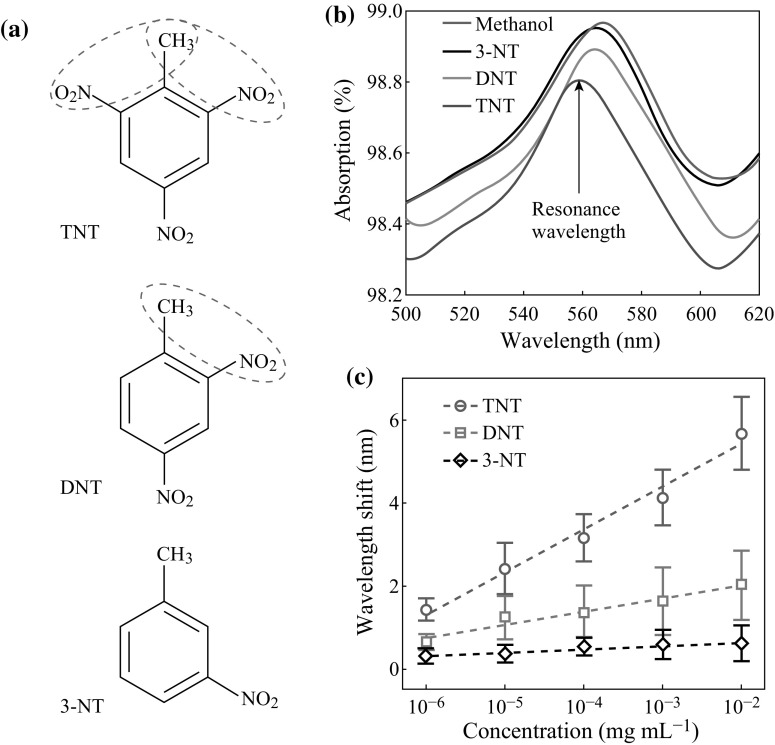
Detections for explosives with the nanoCA. **a** Molecular structures of TNT, DNT, and 3-NT. **b** Absorption spectrum of peptide-modified nanoCA responses to TNT, DNT, 3-NT at 10^−2^ mg mL^−1^ with the pure methanol as control. **c** Dose–response curves of nanoCA which responded to TNT, DNT, and 3-NT in resonance wavelength shift, while compared to the control (mean ± SD, *n* = 20)

The nanobiosensor was designed into TNT specificity in our study. In the measurement, the peptide-modified nanoCA was used to detect TNT at different concentrations with absorption spectrum. DNT and 3-NT, owning similar molecular structures to TNT, were also detected while the solvent of methanol was the control (Fig. 5a). As shown in Fig. 5b, there were significant shifts in resonance wavelength elicited by TNT in absorption spectrum from peptide-modified nanoCA when compared to the control of methanol. However, small wavelength shifts in the resonance peak, even no wavelength shift could be observed in DNT and 3-NT groups. Figure 5c shows statistics for the wavelength shifts of TNT, DNT, and 3-NT groups in absorption spectrum of the nanodevice. For three explosive targets at increasing concentrations, the nanoCA with peptide had a significant dose-dependent behavior to TNT. The wavelength shifts could be increased from around 2 to 6 nm when the concentrations of TNT ranged from 10^−6^ to 10^−2^ mg mL^−1^. However, the device only showed low responses to DNT smaller than 2 nm, while 3-NT almost elicited no response. Moreover, the dose-dependent curve for TNT could be fitted into linear relationship of *y* = 1.018log(*x*) + 7.423, where *x* and *y* represented the concentrations of TNT and their wavelength shifts, respectively. With the fitting curve, the detection limit of the sensor for TNT could be calculated into a low concentration of 3.12 × 10^−7^ mg mL^−1^, equaling approximately 1.37 nM.

**Fig. 6:**
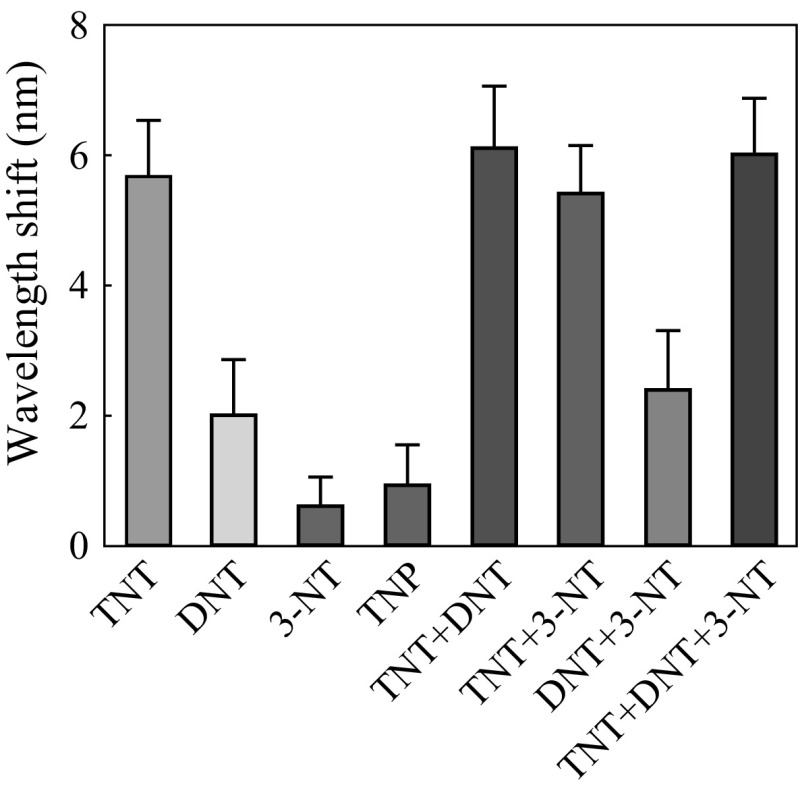
Detection for explosive mixtures with the nanoCA. The concentrations of TNT, DNT, and 3-NT contained in the mixture were fixed on 10^−2^ mg mL^−1^, while solution containing TNT, DNT, 3-NT, and TNP were also 10^−2^ mg mL^−1^ (mean ± SD, *n* = 20)

In military and industry applications, real TNT explosive materials were usually the mixtures of TNT, DNT, and 3-NT. Several chemical compounds, such as DNT, 3-NT, and 2,4,6-trinitrophenol (TNP) had remarkably similar molecular structures to TNT. It was important to verify whether these compounds would interfere with TNT. Thus, the responses of nanocups to DNT, 3-NT, and TNP were recorded, respectively, to study selectivity of the nanosensor for TNT detection. Figure 6 shows that the responses to DNT, 3-NT, and TNP were all smaller than that of TNT, which indicated good selectivity of the nanosensor for TNT. Then, the responses to explosive target mixtures were also measured to further test the selectivity of our nanosensor for TNT. The target compound mixtures of TNT and DNT, TNT and 3-NT, DNT and 3-NT, and the mixture containing TNT, DNT, and 3-NT were employed to test the sensor and compared with the detection for single substance of TNT, DNT, and 3-NT. As shown in Fig. 6, the sensor had significant responses to the mixtures containing TNT, while it provided lower response to the mixture of DNT and 3-NT. The statistics showed that wavelength shifts from mixtures containing TNT were roughly equal to that of detection for TNT alone. It could also be found that the addition of 3-NT had no influence on response to TNT, while the addition of DNT only slightly increased the response. These all indicated that the sensor had a high selectivity to TNT and could not be interfered by other compounds such as DNT and 3-NT.

## Discussion

The last decade studies about biosensors have proposed various high sensitive ways to convert bimolecular bindings into electrical and optical signals [30–33]. Now, the lack of selective bio-components still remained a key challenge, making biosensors inadequate in many applications and preventing their widespread uses. Peptides provided an easy and universal approach to design bio-selectivity with artificial synthesis. However, it was difficult to detect small weight molecules by peptide-based biosensors because of slight structural changes from the specific bindings. Thus, sensor devices had to be used with complex modifications and structures to amplify binding signals. For example, quartz crystal microbalance-based sensor was often used as micro- and nanoparticles to label analyte targets for amplification of gravimetric changes [34, 35]. But, the labels might have an influence on in situ detections for certain analytes. In our study, the LSPR sensor device, nanoCA, provided an excellent platform to quantify interaction between TNT and peptide with high sensitivity, real-time response, and label-free detection. The nanostructured sensor could be combined with peptide to fabricate biosensor for TNT without complex optical coupling and any labels. The binding of TNT to peptide could be monitored selectively by LSPR generated from the periodic nanostructure in transmission measurement. In the measurement, the sensor could detect TNT at concentration as low as 3.12 × 10^−7^ mg mL^−1^. The detection limit was similar to that of several reported works using LSPR of nanomaterials for explosive detections [36–38]. It meant that the sensitivity of nanosensor combining nanocups and the peptides could satisfy explosive detections in practical applications, such as safe checking and environmental monitoring.

Besides sensitivity, the selectivity of the nanosensor was also discussed in our study. The results showed that the sensor had significant responses to TNT, low responses to DNT, and no response to 3-NT and TNP. The nanosensor showed some level of selectivity to TNT versus other chemical molecules that had similar molecular structures. It might be due to the adjacent methyl and nitro groups of molecular structure of TNT and DNT, which had electron interaction with tryptophan and histidine on peptides [39, 40]. Thus, target molecules could be connected to peptides on nanoCA and then elicit resonance wavelength shifts in detections for TNT and DNT, while 3-NT and TNP had no conjunction with the peptide and did not generate shifts in spectrum. The more groups in TNT molecules gave rise to higher affinity to peptides and more significant response signals of the sensor. Uniformly, the nanosensor also showed more significant responses to the mixture containing TNT than that without TNT in the detection for mixed explosive targets. Although the sensor had responses to DNT alone, DNT only increased the response of the sensor slightly in detection for mixture containing TNT and DNT. Those results indicated that the nanosensor could detect TNT in mixtures and would not be interfered with other compounds, even some very similar molecules.

The simple transmission measurement without complex light coupling could help to achieve low-cost and high-throughput detections for explosive compounds. As described above, the nanoCA device was fixed on the bottom of individual well of the 96-well plate, modified with TNT-specific peptides and then stored under 4 °C. Thus, 96 samples containing TNT could be analyzed simultaneously in measurement by using the microplate reader mode of multi-mode detection platform. This microplate-based measurement could not only improve the detection efficiency of the nanosensor with high-throughput characteristics, but also reduce requirement for sample volumes because of microcavities on plates. Moreover, the high-throughput measurement with microplate also provided possibility for array sensing by designing different biofunctions on the surface of nanoCA devices. Indeed, peptides have been reported by several groups to modify sensor devices for bacteria, disease biomarkers, and heavy ions [41–43]. Thus, combining with peptides designed and synthesized into different specificities, the nanobiosensor arrays could be fabricated to detect different chemical and biological molecules in a high-throughput method.

## Conclusion

In summary, TNT-specific peptide was synthesized and used as bio-recognition components for explosive detection of TNT. NanoCA was employed to convert the binding of TNT to the peptide into LSPR responses in transmission spectrum. The nanosensor showed high sensitivity to recognize TNT at the concentration as low as 3.12 × 10^−7^ mg mL^−1^, while keeping selectivity to TNT versus DNT, 3-NT, and TNP with similar molecular structures. Even in the mixture of those three compounds, TNT could also be detected significantly. Combining the nanodevices and specifically designed peptides, this nanobiosensor platform provides a promising approach to develop versatile sensor arrays in further applications for chemical and biological analysis.
